# Network medicine approaches for identification of novel prognostic systems biomarkers and drug candidates for papillary thyroid carcinoma

**DOI:** 10.1111/jcmm.18002

**Published:** 2023-10-19

**Authors:** Medi Kori, Kubra Temiz, Esra Gov

**Affiliations:** ^1^ Faculty of Health Sciences Acibadem Mehmet Ali Aydinlar University İstanbul Türkiye; ^2^ Department of Bioengineering Marmara University İstanbul Türkiye; ^3^ Department of Bioengineering Adana Alparslan Turkes Science and Technology University Adana Türkiye

**Keywords:** co‐expressed module, differential co‐expression network analysis, drug repurposing, papillary thyroid carcinoma, prognostic systems biomarkers, text mining

## Abstract

Papillary thyroid carcinoma (PTC) is one of the most common endocrine carcinomas worldwide and the aetiology of this cancer is still not well understood. Therefore, it remains important to understand the disease mechanism and find prognostic biomarkers and/or drug candidates for PTC. Compared with approaches based on single‐gene assessment, network medicine analysis offers great promise to address this need. Accordingly, in the present study, we performed differential co‐expressed network analysis using five transcriptome datasets in patients with PTC and healthy controls. Following meta‐analysis of the transcriptome datasets, we uncovered common differentially expressed genes (DEGs) for PTC and, using these genes as proxies, found a highly clustered differentially expressed co‐expressed module: a ‘PTC‐module’. Using independent data, we demonstrated the high prognostic capacity of the PTC‐module and designated this module as a prognostic systems biomarker. In addition, using the nodes of the PTC‐module, we performed drug repurposing and text mining analyzes to identify novel drug candidates for the disease. We performed molecular docking simulations, and identified: 4‐demethoxydaunorubicin hydrochloride, AS605240, BRD‐A60245366, ER 27319 maleate, sinensetin, and TWS119 as novel drug candidates whose efficacy was also confirmed by in silico analyzes. Consequently, we have highlighted here the need for differential co‐expression analysis to gain a systems‐level understanding of a complex disease, and we provide candidate prognostic systems biomarker and novel drugs for PTC.

## 1 INTRODUCTION

Recent cancer statistics show that thyroid cancer is the most common endocrine cancer encountered by mankind. In 2022, it was estimated that 43,800 patients were diagnosed with thyroid cancer, and thyroid cancer was responsible for 2230 deaths in the United States.^1^ Papillary, follicular, medullary, and undifferentiated or anaplastic thyroid carcinomas are the major histologic groups of thyroid tumours. Among the various subgroups of thyroid cancer, papillary thyroid carcinoma (PTC) accounts for the largest proportion of all thyroid carcinomas. Notably, the prevalence of PTC is approximately 85% when all thyroid carcinomas are considered.^2^ The aetiology of thyroid cancer is not well understood. Therefore, further studies are needed to uncover the underlying mechanisms of the disease, correct over‐diagnosis, and find prognostic and/or drug candidates to reduce the prevalence of PTC.

Logarithmic advances in high‐throughput sequencing and screening methods in recent decades have produced a substantial amount of X‐ome data at various molecular levels, enabling researchers to perform various bioinformatics approaches for different diseases^3^, ^4^ and also for PTC.^5^ However, some of these studies take only limited account of the reality of the molecular biochemistry of the organism. To discover specific disease biomarkers or drug targets, it is essential to evaluate the entire physical and functional architecture of the organism, because the development of an abnormal phenotype (i.e. disease) is not the result of a single gene, but rather the result of complex gene interactions. One scientific field, network medicine, allows researchers to uncover these complex interactions between biomolecules of a given phenotype in a holistic view.^6^

Gene and protein networks provide valuable data for molecular interactions within the organism, and co‐expression networks represent significantly co‐regulated groups of genes (i.e. modules). Any differentiation in gene correlations between different phenotypes can provide clues to the phenotype and supports the discovery of systems biomarkers.^7^ To reveal co‐expression relationships among genes in thyroid cancer, weighted gene co‐expression network analysis (WGCNA) has been performed in a limited number of studies.^8^, ^9^ WGCNA identifies gene modules by hierarchical clustering. Due to drawbacks of the algorithm, there is still no major standard for WGCNA.^10^ Therefore, in this study, instead of using the WGCNA pipeline, we used a different bioinformatics pipeline whose efficiency has also been demonstrated in various cancers, including ovarian cancer,^11^ cervical cancer,^12^ ovarian cancer stem cells,^13^ and gastric cancer.^14^ To increase the statistical power of our analysis, unlike other studies in this study, we performed a meta‐analysis based on the integration of information from multiple studies.

Accordingly, in the present study, five transcriptome datasets from different cohorts were statistically meta‐analysed to identify differentially expressed genes (DEGs) in PTC. By using culminated common DEGs between datasets, we identified co‐expressed gene pairs that differ between PTC and control phenotypes. After identifying the major significantly co‐expressed gene pairs, we constructed co‐expression networks and determined co‐expressed modules. We evaluated the prognostic capacity of the culminated modules, and the nodes of the modules that have high prognostic capacity were used as proxies to find drug candidates. Text mining and molecular docking analyzes were used to demonstrate the novelty and efficacy of the drug candidates.

## 2 MATERIALS AND METHODS

### 2.1 Selection of transcriptome datasets

Five gene expression datasets from PTC, including GSE60542,^15^ GSE3678, GSE29265, GSE35570^16^ and GSE33630,^17^ were obtained from the Gene Expression Omnibus (GEO)^18^ database. When selecting the datasets, we paid attention to the following three parameters: (i) the selected datasets were from the same platform (i.e. Affymetrix array), (ii) each phenotype contained at least three samples and (iii) the cancer samples belonged to papillary histology. The five datasets obtained included 141 PTC patients and 153 matched non‐tumour controls and were used to identify co‐expressed modules during the study. In addition, the clinical information and expression values of an independent thyroid cancer dataset (THCA) were extracted from The Cancer Genome Atlas (TGCA)^19^ to pre‐clinically validate the prognostic performance of the obtained modules. Since we focus on PTC, we extracted only papillary cancer cases from the THCA‐TCGA, representing a total number of 494 PTC samples.

### 2.2 Identification of DEGs and pathway over‐representation analyses

In this study, we used a well‐established statistical analysis procedure^20^, ^21^ to identify DEGs. Briefly, raw data from each of the five datasets were normalized by calculating the Robust Multi‐Array Average (RMA) expression measure^22^ implemented in the Affy package^23^ of the R/Bioconductor platform (version 4.0.2).^24^ DEGs were identified from normalized expression values using the Linear Models for Microarray Data package (LIMMA).^25^ The Benjamini‐Hochberg method was used to control for false discovery rate (FDR). The adjusted *p* value <0.05 was used as a cut‐off value to determine the statistical significance of the DEGs. To determine the regulatory patterns of DEGs (i.e. up‐ and down‐regulation), the fold‐change threshold was used, with a two‐fold change accepted as statistically significant. Each data set was analysed independently, and results were comparatively analysed to identify common signatures from these independent studies.

Gene set overrepresentation analyses were performed using the functional annotation tool ConsensusPathDB^26^ to reveal associated biological pathways of common DEGs. The analyses preferentially used the Kyoto Encyclopedia of Genes and Genomes (KEGG)^27^ and Reactome^28^ as data sources for the pathways. The p‐values were determined using Fisher's Exact Test, and FDR was applied to control the *p*‐values. An adjusted *p*‐value < 0.05 was considered statistically significant in overrepresentation analyses.

### 2.3 Identification of co‐expressed genes in cancer and control samples

To identify co‐expressed genes between PTC and control samples, an established method^11^ was performed using culminated common DEGs. According to this method, z‐score normalization was performed to eliminate batch effects of each data set, and if the common DEG occurred more than once in the same dataset, the mean expression values were calculated. Then, the correlation patterns of each common gene pair in each condition (i.e. PTC and control) were calculated using correlation coefficients. A cut‐off value for the correlation coefficient was set, corresponding to a *p* value of <0.05 and was used to determine the statistical significance of the pairwise correlations.

### 2.4 Construction of co‐expression networks and determination of differential co‐expressed network modules

To evaluate the major significantly co‐expressed genes, p‐critical values for common DEGs were calculated by integrating the obtained pairwise gene expression correlation values with Spearman correlation coefficients (SCCs). An equation for determining the p‐critical values (Pcritic), which is the cut‐off value of the correlation coefficient, can be found in Equation 1 (Equation 1).

(1) $$p_{\text{critic}}=\text{mean}\;\mathrm{SCC}+1.96*\mathrm{std}\;\text{of}\;\mathrm{SCC}$$

The classification of genes and the significance of expression levels that differ in tumour and non‐tumour samples was determined by the defined threshold condition (parameter Ɛ). This threshold helps to identify differential co‐expression by revealing gene pairs that are significantly linked in the tumour samples but have an opposite direction of correlation in one of the cases. For an equation and consideration of the Ɛ parameter in this study, we used Equation 2 (Equation 2). SCCd represents the correlation coefficient of gene pairs in tumour tissue and SCCh represents the correlation coefficient of gene pairs in healthy tissue.

(2) $$Ɛ=\left|\text{SCCd}-\text{SCCh}\right|>0.5$$

### 2.5 Prognostic performance analysis of differential co‐expressed modules

The prognostic abilities of significant co‐expressed modules were determined using independent RNA‐Seq data from TGCA (THCA‐TGCA).^19^ Clinical information on the samples was collected by TCGA to evaluate the prognostic performance of the modules. Because the most important prognostic factor in all cancers is the stage of the patient, we considered the stage of the patient in the analysis of prognostic performance. Of the 494 PTC samples we obtained from TCGA, 278 samples were stage I, 51 samples were stage II, 110 samples were stage III, 53 samples were stage IV (including IVA and IVC), and the stage was unknown for two samples, so these two samples were not included in the analyses of prognostic performance. The prognostic abilities of the modules were assessed using Kaplan–Meier plots and the log‐rank test. All analyses were performed using the Survival package in R/Bioconductor (version 4.0.2).^24^ Differential co‐expressed modules with a log‐rank *p*‐value of <0.05 were considered statistically significant and accepted as prognostic co‐expressed modules.

### 2.6 Drug repurposing and text mining analysis

Drug repurposing analysis was performed via L1000CDS2: the LINCS L1000 search engine for characteristic directional signatures^29^ using genes within the prognostic gene module. The analysis considered drugs that had an inverse effect on an entered gene expression signature, and the small molecules/drugs with the highest score (i.e. top 50) were selected for further investigation (i.e. text mining and molecular docking analysis).

To determine whether the identified drug candidates had been previously associated with PTC, the identified drug candidates were reviewed in the literature with Python using text mining techniques. Accordingly, the identified drug candidates were searched separately using the following four keywords ‘drug candidate’, ‘drug candidate + thyroid cancer’, ‘drug candidate + thyroid carcinoma’, and ‘drug candidate + cancer’ in the article abstracts and the results were analysed using the BioPython package.^30^ We then, calculated the term frequency (TF) and inverse document frequency (IDF). The TF‐IDF is calculated by multiplying the TF and IDF scores. Drugs with a ‘0’ TF‐IDF score were considered as novel drug candidates in the study.

### 2.7 Molecular docking simulations

The three‐dimensional (3D) crystal structure of the target proteins of the drug candidates was taken from the Protein Data Bank (PDB),^31^ while the structures of the drug candidates were taken from the PubChem database^32^ when available. Molecular docking analyzes were performed using AutoDock Vina software.^33^ For docking simulations, we used previously known binding residues of the target proteins and calculated binding affinities (kcal/mol) to determine binding significance. In addition, the drugs with a higher TF‐IDF value were considered as positive controls. Molecular docking simulations were also performed for these drugs to compare the binding significance of the novel drug candidates.

## 3 RESULTS

### 3.1 Transcriptomic signatures of papillary thyroid cancer: DEGs and their biological insights

**FIGURE 1:**
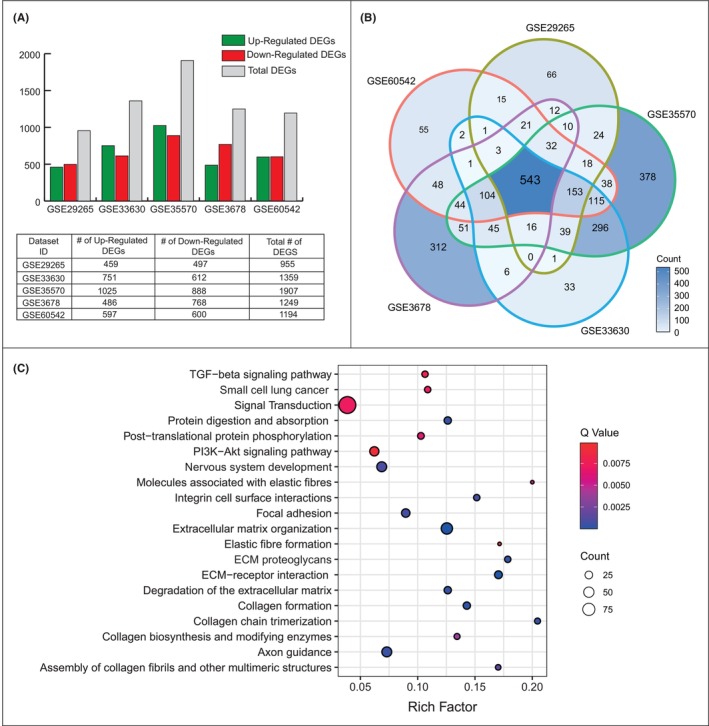
Meta‐analysis of the five transcriptome datasets associated with papillary thyroid cancer (PTC). (A) The number of differentially expressed genes (DEGs) identified for each dataset. (B) Venn diagram showing the common DEGs between all transcriptome datasets. (C) The pathway over‐representation analysis of the common DEGs.

Five microarray gene expression datasets were analysed statistically and individually to identify DEGs. The number of DEGs in each data set showed a wide range from 955 to 1907 genes, and the highest number of DEGs was identified in GSE35570. While the number of up‐regulated genes in datasets GSE33630 and GSE35570 was lower than the number of down‐regulated genes, data sets GSE29265, GSE3678 and GSE60542 showed a profile with a higher number of down‐regulated genes (Figure 1A). Comparative analysis of the resulting DEGs showed that 534 DEGs were common in five transcriptome datasets. To ensure consistency and robustness of the analysis, further analysis was performed using these common DEGs (Figure 1B).

Overrepresentation analysis showed that common DEGs were associated with multiple cancer‐associated pathways (i.e. TGF‐beta pathway, PI3K‐Akt pathway and focal adhesion) and small cell lung cancer. Interestingly, we found that many collagen‐associated pathways, including collagen formation, collagen chain trimerization, collagen biosynthesis and modifying enzymes, collagen fibril assembly and other multimeric structures were significantly associated with PTC. In addition, signal transduction, extracellular matrix organization, axon guidance, and nervous system development were found to be remarkably related to the disease (Figure 1C).

### 3.2 Differential co‐expressed modules for papillary thyroid cancer

**FIGURE 2:**
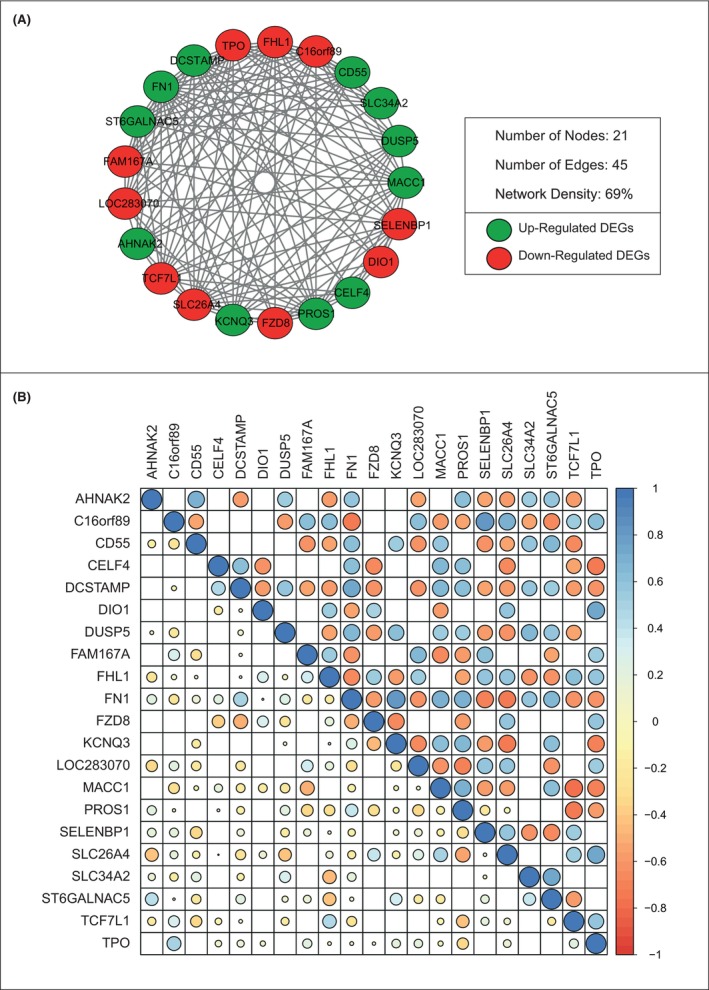
The PTC‐module (A) Co‐expressed differentially expressed genes within the PTC‐module. (B) The correlation plot of the PTC‐module. In the correlation plot the ‘lower triangle’ represents the co‐expression patterns of genes in healthy state, whereas the ‘upper triangle’ represents the co‐expression patterns of genes in diseased state. Correlation coefficient is used to quantify the co‐expression pattern.

After identifying common DEGs in five databases, we used SCCs to identify key gene pairs that are co‐expressed and constructed networks around them. Within the constructed co‐expressed networks, a total of 12 co‐expressed modules were identified. However, considering the parameters we set for detecting highly clustered co‐expressed modules, only one module named ‘PTC‐module’ was identified within the set criteria (Figure 2A).

The PTC‐module consists of 21 nodes with 45 edges and has a network density of 69%. The co‐expression patterns of genes between the control and disease states showed that in the control state, 14 (9.7%) gene patterns were positively correlated and eight (5%) were negatively correlated, whereas in the disease state, 69 (48%) gene patterns were positively correlated, and 73 (51%) gene patterns were negatively correlated, indicating that disease development affects the expression of gene patterns by 83% (Figure 2B).

### 3.3 Prognostic performance of the co‐expressed module

**FIGURE 3:**
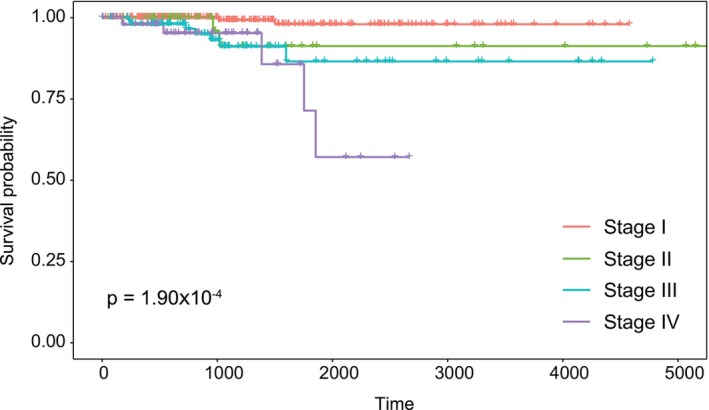
Prognostic performance analyse of the PTC‐module. Kaplan–Meier diagram for estimating patient survival based on stage information with a p‐value.

To pre‐clinically evaluate the prognostic ability of the PTC‐module, an independent RNA‐Seq dataset (THCA‐TCGA) was used, and the prognostic ability of the module was evaluated based on the disease stage of the patients using the Kaplan–Meier plot and the log‐rank test. Since we know that the most important prognostic factor in cancer patients is disease stage, we included this information in the analysis of prognostic performance. The analyzes showed that the prognostic power of the PTC‐module (log‐rank *p*‐value = 1.90 × 10^−4^) was statistically significant, so we found that this module had a high impact on the overall survival of patients according to cancer stage (Figure 3) and was considered as a prognostic systems biomarker in the study.

### 3.4 Identification of novel candidate repurposed therapeutic agents

**FIGURE 4:**
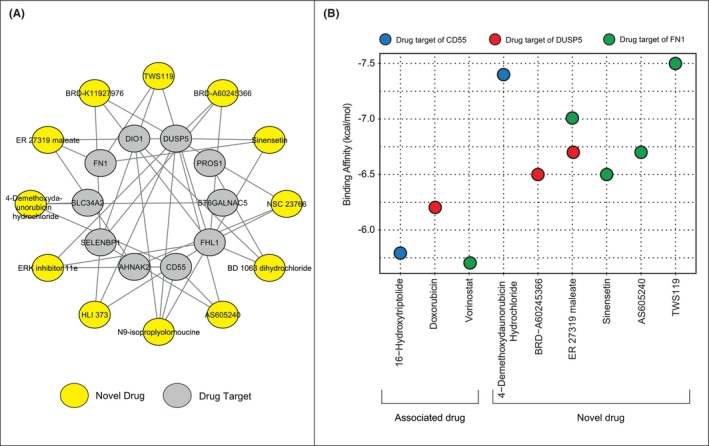
The novel drug candidates and molecular docking simulations. (A) The 12 novel drug candidates and their targets. The novel drug candidates are shown in yellow, while the targets are shown in grey. (B) The bubble plot shows the molecular docking scores of the novel drug candidates. Only the novel drugs that have a significant docking score compared to the drugs that were associated with the disease (i.e. the controls) are shown.

We implemented drug repurposing approach using genes in the PTC‐module using the L100CDS^2^ search engine. Subsequent analysis revealed that, 43 drugs had significant potential to reverse the expression profiles of differentially co‐expressed genes. To reveal the novelty of these drug candidates, the discovered drugs were examined by text‐mining analysis (Table S1). Doxorubicin hydrochloride, doxorubicin and dorsomorphin dihydrochloride yielded the higher TF‐IDF values than the other drug candidates. The drug candidates that have not yet been studied in thyroid cancer (i.e. the TF‐IDF values are ‘0’) were accepted as novel drug candidates for PTC (Figure 4A).

### 3.5 Molecular docking simulations

We performed molecular docking simulations to evaluate the binding affinities of the novel drugs. Of the 12 novel drugs, no 3D‐structure could be found for BD 1063 dihydrochloride, so it was not included in the docking analysis. We examined the 3D structures of PTC‐module proteins targeting 11 novel drug candidates and found suitable structures for the following proteins: (i) CD55 (PDB structure = 1H04),^34^ (ii) DUSP5 (PDB structure = 2G6Z),^35^ and (iii) FN1 (PDB structure = 6XAX).^36^ To specify the significance of affinity binding score, we used drugs with high TF‐IDF value as positive controls, namely 16‐hydroxytriptolide, doxorubicin, and vorinostat. Evaluation of molecular docking simulations revealed that the following drugs 4‐demethoxydaunorubicin hydrochloride targeting CD55 (with a binding affinity of −7.4 kcal/mol), BRD‐A60245366 targeting DUSP5 (with a binding affinity of −6.5 kcal/mol), sinensetin, AS605240, and TWS119 targeting FN1 (with a binding affinity of −6.5 kcal/mol, −6.7 kcal/mol and − 7.5 kcal/mol respectively) and ER 27319 maleate targeting DUSP5 and FN1 (with binding affinities of −6.7 kcal/mol and − 7.0 kcal/mol, respectively) have a significant docking score (kcal/mol) than the controls studied. Therefore, these six drugs represented novel drugs for the treatment of PTC in this study, and their efficiency was also demonstrated by in silico analyzes (Figure 4B).

## 4 DISCUSSION

Thyroid cancer is the most common form of endocrine cancer and PTC is the most common form of thyroid cancer. Since the true risk factors for the disease are unknown, there is still a need to identify prognostic biomarkers and improve existing treatment strategies to improve quality of life and reduce the risk of mortality. In addition, we must remember that biomolecules never function alone, but work together in a complex, interconnected network. Therefore, when discovering biomarkers or drugs, it is important not only to evaluate individual genes, but also to consider the system as a whole and to use the genes taking into account the interactions between them. Accordingly, in this study, we performed bioinformatics meta‐analyzes to identify prognostic system biomarkers and novel drug candidates for PTC.

In our analyzes based on five independent transcriptome datasets, we first attempted to detect differences between the PTC and control phenotypes (i.e. DEG analysis). To increase the reliability of the study and the robustness of the DEG results, we integrated the resulting DEGs in each dataset and focused only on the common 543 DEGs. Analysis of the overrepresentation of common DEGs revealed that several signalling pathways are already associated with thyroid cancer. Indeed, overexpression of TGF‐β1 has been reported to promote PTC cell invasion and migration.^37^ In thyroid carcinogenesis, it became clear that the PI3K/Akt pathway plays an essential role.^38^ In the progression of PTC, deregulation of focal adhesion kinase (FAK) is observed at both post‐transcriptional and translational levels.^39^ Collagen is one of the most abundantly expressed proteins in humans, and collagens are involved at every level of cancer development and establish interactions with cancer cells.^40^ One of the recent studies has shown that collagen family genes (especially COL18A1 overexpression) are associated with poor prognosis and can be used as prognostic biomarkers for PTC.^41^

After finding co‐expressed gene pairs, we created a differential co‐expression network and identified differential co‐expressed gene modules. According to certain criteria, one module, called the ‘PTC‐module,’ was found to be highly clustered and also to have high prognostic significance. When we evaluate the compromising 21 nodes of this PTC‐module, we find that several of these genes have already been individually associated with thyroid cancer, which further strengthens our confidence in our observations when we consider them as systems biomarkers. The following genes: PROS1,^42^ DCSTAMP,^43^ DUSP5,^44^ AHNAK2,^45^ SLC34A2,^46^ FN1,^47^ C16orf89,^48^ FHL1,^49^ TPO,^50^ DIO1^51^ has been associated with thyroid cancer and/or PTC.

In this study, to find novel treatment strategies for PTC, we used PTC‐module nodes as proxies and performed drug repurposing analysis, revealed the novelty of drugs by text mining and demonstrated their efficiency by molecular docking simulations. We identified a total of six novel drug candidates (4‐demethoxydaunorubicin hydrochloride, AS605240, BRD‐A60245366, ER 27319 maleate, sinensetin and TWS119) for the treatment of PTC, whose potential efficacy was also demonstrated by molecular docking simulations. 4‐Demethoxydaunorubicin hydrochloride, also known as idarubicin hydrochloride, is a type of anthracycline antibiotic used to treat acute myeloid leukaemia. To execute its anticancer effects, the drug inhibits topoisomerase II, which this resulting in impaired DNA replication.^52^ A PI3Kγ‐selective inhibitor, AS605240, has shown associations with glomerulonephritis, rheumatoid arthritis,^53^ and Alzheimer's disease^54^ in mouse models. BRD‐A60245366, also known as AS601245, is a JNK inhibitor and has anti‐inflammatory properties. It has been associated with colon cancer. It has been reported that the combined treatment of BRD‐A60245366 and clofibrate has an effect on colon cancer cell lines (i.e. induces cell responses).^55^ ER 27319 maleate is a selective inhibitor of Syk kinase and has been associated with head and neck cancer.^56^ Sinensetin has multiple pharmacological activities, including antineoplastic, anti‐inflammatory and antimicrobial activities. It has been associated with gastric, colon, and breast cancer.^57^ TWS119 is a potent inhibitor of GSK‐3β and it has been reported that TWS119 increases the replication and cytolytic activity of human γδT cells against human colon cancer cells. These γδT cells represent unique T cell subpopulations and can be used in cancer immunotherapy. Therefore, TWS119 has been used as a complementary agent to enhance γδT‐cell‐based immunotherapy for colorectal cancer.^58^

In conclusion, the distribution of possible significant changes in gene pairs during the transition from a normal to a tumour phenotype is of great importance. Moreover, the evaluation of the modular structures around these co‐expressed gene pairs will provide us with much more effective results in the development of diagnostic, prognostic biomarkers, and/or treatment strategies. Therefore, in this study, we investigated the different co‐expression profiles between PTC and control phenotypes. Based on this information, we proposed a module (i.e. the PTC‐module) with a high prognostic capacity and suggested it as a system biomarker. In addition, six novel drug candidates for PTC were presented in this study, which deserve to be investigated with experimental studies. The benefits of integrative bioinformatics approaches are also highlighted to develop a network medicine approach for thyroid cancer prognosis and treatment in the future.

## AUTHOR CONTRIBUTIONS

**Medi Kori:** Data curation (equal); formal analysis (equal); methodology (equal); writing – original draft (equal); writing – review and editing (equal). **Kubra Temiz:** Data curation (equal); formal analysis (equal); writing – original draft (equal). **Esra Gov:** Conceptualization (equal); methodology (equal); supervision (equal); writing – review and editing (equal).

## CONFLICT OF INTEREST STATEMENT

The authors confirm that there are no conflicts of interest.

## Supporting information


**Table S1.** Repurposed drugs and text mining results of candidates.

## Data Availability

The data that support the findings of this study are available from the corresponding author upon reasonable request.
